# Ribosomal stress-surveillance: three pathways is a magic number

**DOI:** 10.1093/nar/gkaa757

**Published:** 2020-09-17

**Authors:** Anna Constance Vind, Aitana Victoria Genzor, Simon Bekker-Jensen

**Affiliations:** Center for Healthy Aging, Department of Cellular and Molecular Medicine, University of Copenhagen, Blegdamsvej 3B, DK-2200 Copenhagen, Denmark; Center for Healthy Aging, Department of Cellular and Molecular Medicine, University of Copenhagen, Blegdamsvej 3B, DK-2200 Copenhagen, Denmark; Center for Healthy Aging, Department of Cellular and Molecular Medicine, University of Copenhagen, Blegdamsvej 3B, DK-2200 Copenhagen, Denmark

## Abstract

Cells rely on stress response pathways to uphold cellular homeostasis and limit the negative effects of harmful environmental stimuli. The stress- and mitogen-activated protein (MAP) kinases, p38 and JNK, are at the nexus of numerous stress responses, among these the ribotoxic stress response (RSR). Ribosomal impairment is detrimental to cell function as it disrupts protein synthesis, increase inflammatory signaling and, if unresolved, lead to cell death. In this review, we offer a general overview of the three main translation surveillance pathways; the RSR, Ribosome-associated Quality Control (RQC) and the Integrated Stress Response (ISR). We highlight recent advances made in defining activation mechanisms for these pathways and discuss their commonalities and differences. Finally, we reflect on the physiological role of the RSR and consider the therapeutic potential of targeting the sensing kinase ZAKα for treatment of ribotoxin exposure.

## INTRODUCTION

Stress responses are evolutionarily conserved signaling pathways that have evolved to cope with detrimental cellular perturbations. As such, the cell's ability to rapidly sense and adequately respond to extra- and intra-cellular signals is crucial for maintaining cellular integrity. Such signals include hormones and growth factors, which are important for regulating cell physiology, but also agents of a more harmful nature such as toxins, infections, byproducts of metabolism, and ultra violet (UV)-radiation. Depending on the nature, duration and severity of stress signals, cells initiate stress responses that serve to curtail the negative consequences of disrupted homeostasis.

**Figure 1.**
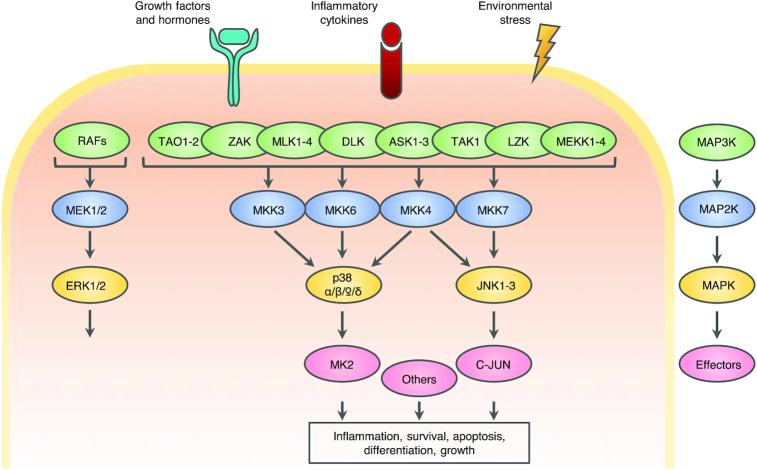
The MAP kinase signaling network. (**A**) A vast array of stimuli can activate the MAP kinase network, including environmental stressors, inflammatory cytokines, growth factors and hormones. The canonical activation pattern of MAPK cascades begins with the stimulus-specific activation of a MAP kinase kinase kinase (MAP3K, green) that activates MAP kinase kinases (MAP2K, blue) that activates MAP kinases (MAPK, yellow). The cellular effects of MAP kinase signaling is mediated through the phosphorylation of a vast amount of effector proteins.

MAP kinase cascades are key signal transduction pathways involved in the coordination of numerous stress responses (Figure 1). This large kinase network can regulate a plethora of biological processes in response to intra- and extracellular stimuli. In general, MAP kinases are organized with a three-tier architecture starting with MAP kinase kinase kinases (MAP3Ks) that activate MAP kinase kinases (MAP2Ks) that in turn activate MAP kinases. This molecular hierarchy is highly conserved throughout evolution, and many of the kinases have homologs in other organisms, demonstrating their importance in maintaining homeostasis on a cellular level.

The proto-typical MAP kinases comprise three groups; Extracellular signaling-Regulated Kinase (ERK1 and 2), c-Jun N-terminal Kinase (JNK1, 2 and 3) and p38 (p38α, β, γ and δ). In addition, several atypical MAP kinases exist, denoted ERK3,4,5 and 7 and Nemo-Like Kinase (NLK). The three classes of typical MAP kinases respond to multiple cellular cues, and all require dual phosphorylation in their activation loop to achieve maximum enzymatic activity. The relevant region contains a Thr-X-Tyr motif, where both threonine and tyrosine must be phosphorylated for activation. X denotes Pro, Glu and Gly for JNKs, ERKs, and p38s respectively (1).

Members of the ERK MAP kinase family primarily respond to cytokines, hormones and growth factors. In contrast, the p38 and JNK signaling pathways are often referred to as the Stress-Activated Protein (SAP) kinases as they are activated in response to various exogenous and endogenous stress stimuli. These include environmental stressors, such as reactive oxygen species (ROS), osmolarity changes, toxins and UV-radiation, but also proinflammatory cytokines and bacterial lipopolysaccharide (LPS). Activation of the SAP kinases mediates cell-fate decisions, promoting both pro-survival and pro-apoptotic signaling, and modulate immune responses by controlling the expression of inflammatory cytokines (2,3). The importance of an appropriate activation of these kinases under diverse stresses is highlighted by the fact that dysregulation of SAP kinase signaling is associated with numerous pathologies, including neurodegenerative disorders, inflammatory diseases and cancer (2,4–6)

In this review, we summarize our knowledge on how p38 and JNK are activated upon ribosomal impairment, and the ensuing biological consequences. Ribosomal poisons in the form of ribotoxins, antibiotics and UV radiation interrupt translational elongation and elicit the RSR. In contrast to other ribosomal surveillance mechanisms such as Ribosomal Quality Control (RQC) and the Integrated Stress Response (ISR), the RSR elicits a proinflammatory cascade controlled by MAP kinase signaling.

## THE p38 SIGNALING PATHWAY

There are four members of the p38 family, p38α/*MAPK14*, p38β/*MAPK11*, p38γ/*MAPK12* and p38δ/*MAPK13*. They are encoded by four separate genes that give rise to a number of alternatively spliced constructs, and can further be divided into two groups based on their sequence homology: p38α/β and p38γ/δ (7). p38α and p38β are both ubiquitously expressed, whereas p38γ and p38δ are expressed in a cell type and tissue-specific manner, possibly reflecting a more specialized function. p38γ is mainly found in skeletal muscle, while p38δ is significantly expressed in pancreas, kidney, testis and small intestine (7). Knockout of p38α is embryonic lethal due to placental defects (8–10), but p38β-, p38γ- and p38δ-deficient mice are viable and fertile and present with minimal phenotypes (7). As an example, knockout of p38γ revealed that this isoform is important for endurance exercise-induced biogenesis of mitochondria, consistent with its marked overrepresentation in skeletal muscle (11). Furthermore, and in accordance with p38δ’s tissue-restricted expression pattern, p38δ knockout mice display a higher glucose tolerance due to increased insulin secretion from pancreatic β-cells (12).

The canonical activation mechanism for p38 is by dual phosphorylation of the Thr-Gly-Tyr motif by the MAP2Ks MKK3 and MKK6 (13–15). Additionally, the JNK-activating MAP2K MKK4 is also able to activate p38α under certain conditions (16). MKK3 and MKK6 appear to serve functionally redundant roles, and mice deficient for either MKK3 or MKK6 are viable and fertile although displaying reduced cytokine production (17–19). On the other hand, inactivation of both genes confers embryonic lethality, and embryos present with many of the same symptoms as p38α knockouts, including placental defects (16). Upstream of the MAP2K layer, several MAP3Ks exert control over p38 activation, and this subject is well treated in other reviews (20,21). Non-canonical modes of p38 activation have also been reported. Here, TAB1 (transforming growth factor-β-activated protein kinase 1-binding protein) mediates activation of p38α in the absence of upstream kinase signaling by promoting cis-autophosphorylation of the activation loop (22).

p38 was first identified as a protein with a critical role in production of the proinflammatory cytokines IL-1 and TNFα in response to LPS-stimulation of monocytes (23). It has since become apparent that p38 plays a broad role in immune signaling, as its activation results in stabilization and translation of multiple cytokine mRNA (7). Studies employing chemical inhibitors and genetic inactivation have led to the identification of numerous physiological targets of p38. These include components of the AP-1 transcription factors complex (e.g. ATF2, Fos), Ser/Thr kinases (e.g. MK2) and RNA binding proteins (e.g. HuR), as well an expanding list of regulatory, membrane and structural proteins (24). p38 is a proline-directed kinase, the substrate specificity of which is influenced by interactions between docking sites on the substrate and interaction motifs on the kinase. Albeit the four members of the p38 family have many overlapping targets and some functional redundancy, there are notable differences. For instance, while MK2 is a prominent substrate for p38α/β, this kinase is phosphorylated to a lesser degree by p38γ/δ (25,26). Additionally, small molecule kinase inhibitors such as SB203580 and SB202190, which have contributed greatly to our understanding of p38 signaling pathways, only inhibit p38α and p38β, while others like BIRB796 inhibit all four isoforms (27).

## THE JNK SIGNALING PATHWAY

Three genes encode JNK kinases, JNK1/*MAPK8*, JNK2/*MAPK9* and JNK3/*MAPK10*, collectively giving rise to at least 10 different isoforms by alternative splicing (28). While JNK1 and JNK2 are universally expressed, JNK3 is largely restricted to testis and neuronal tissue (29). Mice lacking any of the three isoforms are viable, as is also the case for double knockout of JNK1/3 and JNK2/3 (29–31). However, simultaneous knockout of JNK1 and JNK2 confers early embryonic lethality due to defective neural tube closing and dysregulated apoptosis in the brain (30,31). Such data suggest that JNK1 and JNK2 are largely functionally redundant kinases and highlights the importance of JNK signaling in the nervous system.

JNK kinases are activated by many of the same cellular stresses and MAP3Ks as the p38 module, but instead of MKK3 and MKK6, JNK activity is controlled by the MAP2Ks, MKK4 and MKK7 (32). Unlike the case for MKK3 and MKK6, these two MAP2Ks exhibit non-redundant roles, as MKK4 preferentially phosphorylates Tyr, whereas MKK7 generally phosphorylates Thr in JNK’s Thr-Pro-Tyr activation loop (32). Since JNK as a typical MAP kinase requires dual phosphorylation on Tyr and Thr for activation, it has been suggested that MKK4 and MKK7 work synergistically to achieve downstream kinase activation. In support of this, mice lacking either MKK4 or MKK7 succumb before birth presumably due to the inability of these kinases to functionally compensate for each other (29,33–35). Nevertheless, such a synergistic relationship is not fully consistent with the fact that MKK4 and MKK7 exhibit a distinct pattern of tissue enrichment and subcellular localization (36,37), and by the fact that *mkk4-/-* and *mkk7-/-* embryos display different phenotypic anomalies (33,34,38,39). Binary interaction between MAP3Ks and the corresponding MAP2Ks and MAPKs represent one determinant of selective MAPK pathway activation. Further specificity can be conferred by scaffolding and adaptor proteins. Such proteins stabilize and spatially restrict the direct interactions between MAP kinase signaling components. Examples of well-studied MAP kinase scaffold factors are the JNK-interacting proteins (JIPs) which are required for optimal JNK activation in a variety of settings (40).

JNK was originally identified in 1993 as the kinase that phosphorylated the transcription factor c-Jun in response to UV light and expression of transforming oncogenes (41,42). Phosphorylated c-Jun promotes formation of the AP-1 transcription factor complex and thus activates genes containing AP-1 binding sites. These include cytokine genes and genes that control cell cycle, survival and apoptosis (43–45). Substrates for JNKs also include other transcription factors, microtubule-associated proteins (MAPs) and members of the apoptosis-regulating BCL2 protein family (e.g. BAD and BAX) (46).

In sum, a plethora of intra- and extracellular signals trigger activation of the SAP kinases p38 and JNK. The context-specific dosing of downstream phosphorylation events determine the balance between cellular outcomes such as survival, cell death, differentiation and inflammatory signaling.

## MAP KINASE ACTIVATION IN RESPONSE TO RIBOTOXIC STRESS

**Figure 2.**
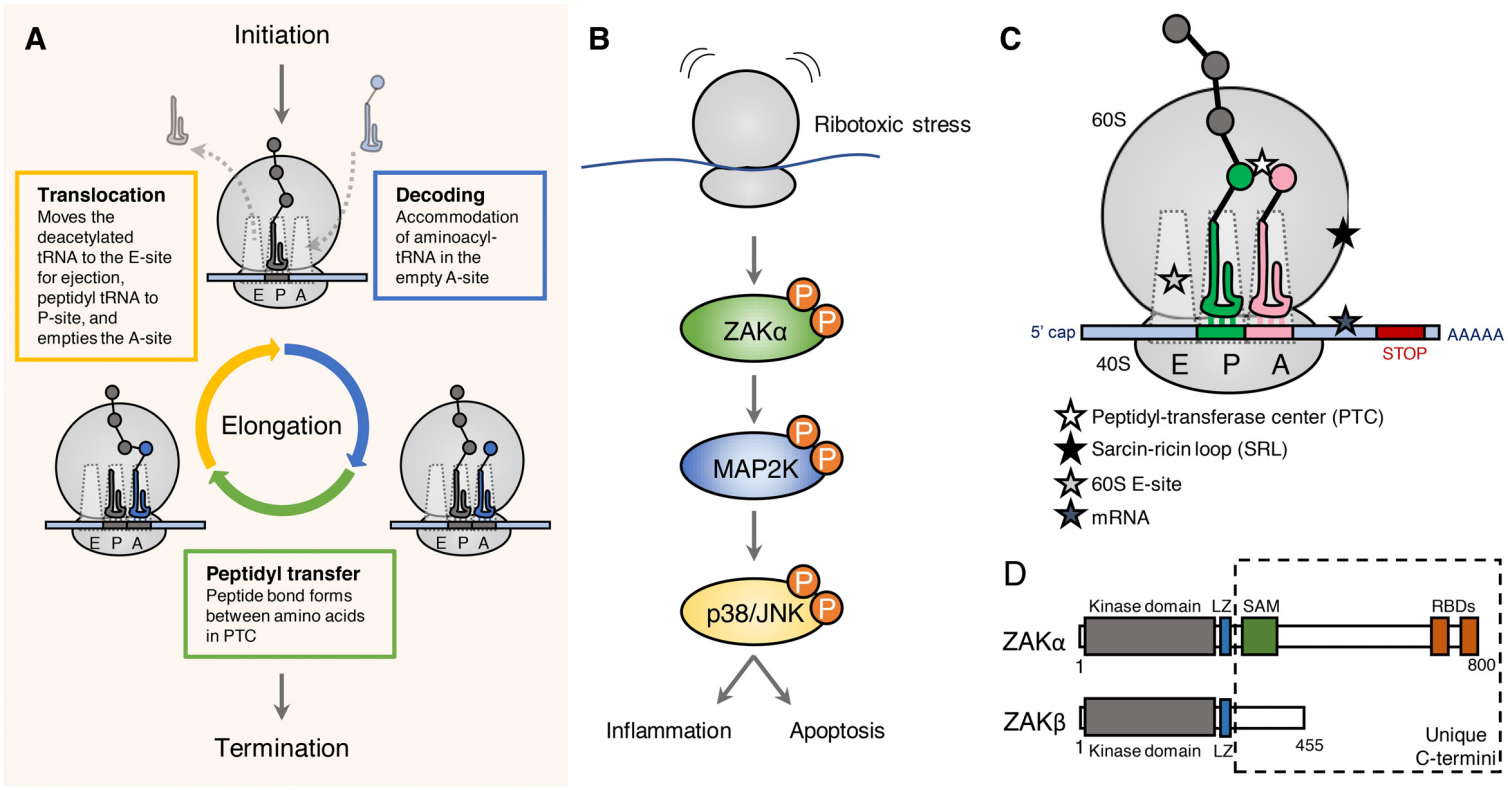
The translation elongation cycle and the ribotoxic stress response. (**A**) The ribosome can decode messenger RNA (mRNA) and translate it into protein in three sequential events; initiation, elongation and termination. Elongation is a three-step cycle that is repeated until the protein is fully synthesized, and the stop codon is reached. The key processive reactions are (i) the capture of amino acid loaded tRNA, (ii) transfer of the amino acid to the growing polypeptide chain and (iii) the release of vacant tRNA, which occur in the ribosomal A-, P- and E-sites, respectively. (**B**) Ribotoxic stress is sensed by the MAP3K ZAKα leading to activation of MAPKs p38 and JNK and inflammatory signaling. In case of strong and sustained signaling the cell will undergo regulated cell death. (**C**) Overview of ribosomal insults known to activate the RSR – see also Table 1. (**D**) Alternative splicing of the *ZAK* gene results in two different MAP3Ks, α and β, that share the first 11 exons followed by unique exons. ZAKα is an 800 amino acid protein containing a kinase domain, a leucine zipper (LZ), a sterile-alpha motif (SAM) and two ribosome-binding domains (RBDs). ZAKβ is a 455 amino acid protein that is identical to ZAKα in its N-terminus but has a unique C-terminus.

Given the essential role of protein synthesis (Figure 2A) in all living cells, it is not surprising that stress response pathways have evolved to detect and counteract physiological and pathological obstructions to translation. One such pathway is the RSR, which is a MAP kinase-mediated proinflammatory signaling cascade that is mounted in response to different ribosomal insults (Figure 2B). The term ‘ribotoxic stress response’ was coined in 1997 by Iordanov *et al.* The authors demonstrated that p38 and JNK was activated by drugs and toxins that interfere with the region of 28S rRNA critical for aa-tRNA binding, peptidyl transfer and translocation (47). Since then additional triggers of the RSR has been identified, and they can roughly be subdivided into translation inhibitors (e.g. anisomycin and cycloheximide), ribotoxins (e.g. ricin, Shiga toxin and α-sarcin), chemotherapeutics (e.g. doxorubicin) and UV-radiation (Figure 2C, Table 1). These agents all cause structural damage to rRNA and impair ribosome function, and lead to strong activation of p38 and JNK. Such signaling initially triggers a proinflammatory response, but a sufficient duration or magnitude of RSR signaling will eventually cause cell death (48–51).

**Table 1.** Known ribotoxic stress agents and their mechanism of action

	Ribosomal target	Cellular effect
Translation inhibitors		
Anisomycin	Prevents peptide bond formation at PTC, 60S A-site (52) Interacts with domain V of 28S rRNA	p38 and JNK activation (47) in a ZAKα dependent manner (66,70,72) and leads to transcription and secretion of IL-8 via ZAK/p38 signaling axis (48,71)
Cycloheximide	Inhibits translocation via interactions with the 60S E-site	p38 and JNK activation in a ZAKα dependent manner (47,72)
Lactimidomycin	Blocks initiation by inhibiting first round of translocation at the 60S E-site (52)	p38 and JNK activation in a ZAKα dependent manner (72)
Harringtonine	Blocks initiation by inhibiting first peptide bond formation at PTC, 60S A-site (153)	p38 and JNK activation in a ZAKα dependent manner (72)
Deoxynivalenol / trichothecene toxins	Prevents peptide bond formation at PTC, 60S A-site	p38 and JNK activation (154)
Onnamide A	Inhibits protein translation measured by [^3^H]-leucine incorporation (62)	p38 and JNK activation (62)
Theopederin B	Inhibits protein translation measured by [^3^H]-leucine incorporation (62)	p38 and JNK activation (62)
Ribotoxins		
Ricin *Ricinus communis*	Depurination of adenine in the sarcin/ricin loop located in domain VI of 28S rRNA (155)	p38 and JNK activation and IL8 production in a ZAKα dependent manner (47,71,72,77) Ricin-induced cell death is mediated by ZAK (71) *Zak-/-* mice have lower duodenal pathology score than *wt* mice following oral ricin intoxication (77)
Shiga toxin *Shigella dysenteriae Escherichia Coli*	Depurination of adenine in the sarcin/ricin loop located in domain VI of 28S rRNA (155)	p38 and JNK activation (50) in a ZAK dependent manner (71) *N*-glycosidase activity of Stx1 is required for p38 and JNK activation (49) Stx at high doses induce cell death via p38 and ZAK (49,71)
α-sarcin *Aspergillus giganteus*	Cleavage of phosphodiester bond in the sarcin/ricin loop (53)	p38 and JNK activation (47)
Chemotherapeutics		
Doxorubicin	Inhibits protein translation measured by [^3^H]-leucine incorporation (61)	p38 and JNK activation and cell death in a ZAK dependent manner (61,94) Upregulation of transcription and secretion of inflammatory cytokines IL-6 and CXCL1 via p38/ZAK (58,94)
Daunorubicin	Structurally similar to doxorubicin (61)	p38 and JNK activation and cell death in a ZAK dependent manner (61)
UV-radiation		
UV-B and UV-C	Induce rRNA lesions at domain V and VI of 28S rRNA (63)	p38 and JNK activation (63) in a ZAKα dependent manner (66,70) Sorafenib, a multi-kinase inhibitor, repress UV-induced apoptosis via ZAK and JNK (76)
UV-damaged mRNA		Activation of ZAKα, p38 and JNK in cells after transfection with UV-irradiated mRNA (66)

### Ribotoxic stress-inducing agents

Anisomycin is an antibiotic that targets the peptidyl transferase center (PTC), where the peptide bonds are formed during protein synthesis (52). Other translation inhibitors, like cycloheximide, occupy the ribosomal E-site and blocks the ribosomal translocation step of elongation (52). In support of their hypothesis that interference with 28S rRNA is the trigger of the RSR, Iordanov *et al.* also achieved strong activation of the RSR by administration of ribotoxins (Shiga, ricin and α-sarcin). These enzymes all exert their cytotoxic function through modification of the sarcin/ricin loop (SRL) located on the 28S rRNA (47). Shiga is produced by the dysentery-causing bacterium *Shigella dysenteriae* and Shiga-like toxins (Stx) are produced by some pathogenic strains of *Escherichia coli*. Ricin is a plant-derived glycoprotein isolated from castor beans (*Ricinus communis*), while α-sarcin is an RNA endonuclease from the fungus *Aspergillus giganteus*. Ricin and Shiga toxins both hydrolyze the N-glycosidic bond of a conserved adenine residue in the SRL, whereas α-sarcin cleaves the phosphodiester bond adjacent to this adenine residue (53,54). The SRL is critical for delivery of amino acid-bound tRNA and elongation factors to the translational pocket of the ribosome. In addition, the SRL plays a critical role in stimulating the GTPase activity and subsequent release of elongation factors from the ribosome (55–57).

MAP kinase-regulated production of proinflammatory cytokines are common responses to a range of chemotherapeutics (58,59) Such signaling is widely believed to be a significant cause of detrimental side effects to cancer treatment. Doxorubicin is an anthracycline widely used in the treatment of hematological and solid tumors, and it is known to induce p38 and JNK activation (58,60). Sauter *et al.* reported that treatment of cells with high doses of doxorubicin activates the RSR in keratinocytes leading to inflammatory signaling and apoptosis (61). An argument for the ribosome being the relevant target was supported by the finding that doxorubicin at the same doses strongly inhibited protein translation. In addition, pre-treatment of cells with the ribosome inhibitor emetine, which by itself does not activate RSR, blocked doxorubicin-induced activation of p38 and JNK. Since then, several other chemotherapeutic agents have been shown to inhibit the ribosome and activate RSR signaling. These include daunorubicin, another anthracycline-class drug (61), and Onnamide A and Theopederin B, two natural compounds related to mycalamides (62).

Finally, UV light has also been shown to be a ribotoxic stress-inducing agent (63). Even in the absence of DNA, UV-light is a potent inducer of MAPK activation. Studies with enucleated HeLa cells demonstrated a nucleus-independent activation of JNK after UV irradiation (64). This extranuclear response has been suggested to be mediated by UV-induced inactivation of protein tyrosine phosphatases and the resulting increase in tyrosine kinase activities (65) or by ribotoxic stress (47,63). In support of the latter hypothesis, Iordonov *et al.* concluded that UV-irradiation introduces ribosomal photodamage and inhibits ribosome function (63). In primer extension reactions, the authors detected UV-induced nucleotide damage in the 3′end of 28S rRNA. These lesions occurred mainly at adjacent pyrimidine nucleotides, suggesting that they consist of pyrimidine dimers, similar to photoproducts induced by UV light in DNA. Some of these lesions affected two critical regions of 28S rRNA; domain V, containing the peptidyl transferase ring, and domain VI, containing the SRL (63). As previously mentioned, these rRNA structures are also the relevant targets of several ribotoxic stress agents, including anisomycin and ribotoxic enzymes (47). More recent work has shown that the RSR is also elicited by translation arrest on a UV-damaged mRNA template (66). Whether it is a combination of damage to rRNA and mRNA, or only the latter, that activates the RSR after UV-irradiation remains to be clarified.

### Activation of the ribotoxic stress response

The RSR is a MAP kinase signaling cascade mounted in response to defective ribosomes. Several upstream kinases have been implicated in the activation of this response including the double-stranded RNA-dependent Protein Kinase (PKR) (67,68), HCK (69) and the sterile-motif alpha and leucine zipper kinase (ZAK) (70–72). One group found that PKR is partially needed for complete MAPK activation in response to the RSR activators anisomycin, deoxynivalenol and ricin (67,68). However, a study using MEFs from PKR knockout mice did not support a role for PKR in activation of p38 and JNK in response to anisomycin or UV (73). Similarly, evidence for HCK playing an important role in the response is limited.

ZAK, also known as MAP3K20 or MLTK, is a MAP3K belonging to the MLK subfamily. Two major splice variants of ZAK exist; ZAKα (800 amino acids) and ZAKβ (455 amino acids). Originally, overexpression of these proteins were found to promote apoptosis in a hepatoma cell line (74), and ZAK was also reported to be activated by osmotic stress, causing disruption of actin fibers and altered cell morphology (75). Since then multiple functions of ZAK have been proposed, including the activation p38 and JNK in response to ribotoxic stress (61,70,72,76). This was first described in 2005 by Wang *et al.* through use of the ZAK inhibitor dihydro-pyrrolopyrazole quinoline (DHP-2) and by siRNA-mediated knockdown (70). These authors further showed that ZAK mediates p38 and JNK activation in COS-7 cells exposed to anisomycin and UV, but not the inflammatory cytokines TNFα and IL-1β.

An activating role for ZAK also in ribotoxin-induced RSR was demonstrated in 2008 by Jandhyala *et al.* They showed that both chemical inhibition and siRNA-mediated knockdown of ZAK prevented MAP kinase activation in response to ricin and Stx (71), which was later corroborated in ZAK knockout cells (72). Later, the same group showed that bone-marrow derived macrophages established from *zak*-/- mice were completely deficient for p38 and JNK activation upon ricin exposure (77). ZAK also appears to be the relevant upstream MAP3K when cells encounter other sources of ribotoxic stress. Thus, ablation of ZAK activity, both through chemical inhibition with sorafenib and nilotinib and siRNA-mediated knockdown suppressed doxorubicin-induced activation of p38 and JNK (61). In addition, knockdown of ZAK suppressed UV-induced MAP kinase activation (70), which was recently validated in ZAK knockout cells (66). Taken together, an overwhelming body of literature using diverse sources of ribotoxic stress places ZAK at the top of the MAP kinase signaling cascade that constitutes the RSR.

### Sensing ribotoxic insults

Despite the emergence of ZAK as a key upstream regulator of RSR signaling, it is not fully understood how cells sense and communicate the presence of structurally diverse ribotoxic insults towards p38 and JNK activation. The underlying activation signal(s) does not appear to constitute mere translation arrest, as several translation inhibitors, such as emetine, pactamycin, and puromycin, are unable to (or only weakly) trigger the response (47). Additionally, toxins that inactivate the elongation factor EEF2 do not initiate RSR (47,78). A recent study by our group showed that the long isoform of ZAK, ZAKα, constitutes a molecular sensor of ribotoxic stress by virtue of two C-terminal ribosome binding domains (72) (Figure 2D). The shorter ZAKβ isoform does not contain such domains and does not support RSR signaling. Instead its C-terminus is encoded for by an alternative exon, and this protein is likely to mediate MAP kinase activation in response to other and presently unknown cellular insults. By inserting its flexible C terminus into the inter-subunit space, ZAKα is placed in a unique position to probe structural conformations of the translationally active pocket of the ribosome (72). This direct communication with the ribosome allows ZAKα kinase activation in the presence of functionally diverse ribosome inhibitors, structural rRNA damage induced by ribotoxins and presumably UV-induced photo-products. ZAKα’s signature Sterile Alpha Motif (SAM) domain likely plays a key role in harnessing kinase activity, as mutation of this domain was shown to confer constitutive activation (72) and cause a developmental bone/limb disorder in humans and mice (79).

### Pathological implications of the Ribotoxic Stress Response

The primary outcome of ZAKα-induced RSR signaling elicits activation of SAPKs p38 and JNK. In addition to controlling cell-fate decisions such as cell death and differentiation, these kinases activate inflammatory signaling by virtue of their control over cytokine expression (3,6). In addition, it has been shown that RSR signaling in concert with the Unfolded Protein Response (UPR) regulates Bcl-2 to balance apoptosis and cell survival in leukocytes during immune responses (80–82). The ability of the RSR to activate both pro-inflammatory and pro-apoptotic pathways has motivated the proposal of pathway inhibition as a therapeutic strategy to combat ribotoxin intoxication (83,84). It is likely that RSR signaling contributes to the pathology of the gastrointestinal and renal complications associated with infection with Stx-producing *E. coli* (STEC). This group of food-borne pathogens can cause severe disease in humans including hemorrhagic colitis and, in susceptible patients, hemolytic uremic syndrome (HUS). HUS is a triad of hemolytic anemia, thrombocytopenia, and acute kidney failure. In support of this notion, mice that are unable to produce TNFα from macrophages survive Stx2 intoxication longer compared to WT mice (85). Additionally, injection of TNFα increases the mortality of mice intoxicated with sub-lethal doses of Stx1. This enhanced phenotype was associated with exacerbated glomerular damage, decreased kidney function, and increased renal apoptosis (86). Several studies also support that RSR-driven MAP kinase signaling boosts cytokine production upon Stx exposure (49,71,87,88).

p38 and JNK are both attractive drug targets for a number of diseases, including the potentially lethal Stx-induced gastrointestinal and renal damage caused by STEC infection. However, the many and pleiotropic roles of these kinases have so far prevented successful clinical trials with chemical inhibitors (89,90). Instead, inhibition of upstream and signal-specific activator kinases may represent a viable approach (83,89). This strategy has already been pursued with some success with inhibition of the MAP3K ASK1 for the treatment of nonalcoholic steatohepatitis (91). In the case of ribotoxin exposure, inhibition of ZAK could similarly represent a promising therapeutic strategy. Indeed, a couple of studies suggest that RSR signaling contributes to the pathogeneses observed after ribotoxin exposure (77,83). Jandhyala *et al.* reported that ZAK knockout mice suffer less duodenal damage upon ricin exposure, likely due to attenuation of cytokine-mediated tissue destruction (77). Other supporting evidence was obtained in rabbits exposed to Stx (83). Here, ZAK inhibition by the multiple-kinase inhibitor imatinib decreased heterophil infiltration and colonic inflammation (83). In spite of these encouragements, attempts to exploit our knowledge of RSR signaling therapeutically have so far been very limited. Further pre-clinical studies are required to determine how much of the detrimental organismal effects of ribotoxin exposure can be attributed to RSR signaling (84,92).

The RSR is also activated by a subset of often used anti-cancer drugs. This signaling may be linked to some of the detrimental side effects of chemotherapy including heart failure (59). For example, ribosome inhibition and ensuing p38 signaling has been suggested to be involved in doxorubicin-induced cardiomyopathy (60,61). MAP kinase inhibition has been shown to effectively reduce apoptosis of cardiomyocytes after doxorubicin and daunorubicin treatment (60,93). Inhibition of p38 in macrophages was also shown to reduce doxorubicin-induced proinflammatory responses, without compromising the anti-proliferative effects of the drug in a cancer cell line (58). Doxorubicin caused activation of p38 and JNK in a ZAK-dependent manner in both keratinocytes and monocytes (61,94), and in keratinocytes ZAK inhibition also effectively blocked apoptosis. Notably, this effect was not observed for cancerous HeLa cells (61). Time will tell whether there is a rationale for the use of ZAK inhibition in combination with RSR-activating chemotherapy (61,94).

In cultured cells, UV-irradiation is another potent trigger of RSR activation. This does not appear to be dependent on DNA damage, and human skin fibroblasts exhibit an almost 7-fold higher amount of oxidized RNA compared to DNA (95). It intuitively follows that the RSR could play an important physiological role in the skin's response to UV light. Melanoma patients treated with the BRAF inhibitor sorafenib are in high risk of developing UV-induced squamous cell carcinoma during the course of their treatment. (96,97). Sorafenib is known to have strong off-target effect against ZAK (61), and was reported to suppress UV-induced and ZAK-dependent apoptosis in cultured keratinocytes (76). These results suggest that ZAK and RSR signaling has an anti-tumorigenic role in skin. Defining the role(s) of the RSR in UV-irradiated skin will be an important future challenge, both with regards to elucidation of the physiological roles of the RSR and potential new insights into skin cancer biology.

## OTHER RIBOSOMAL SURVEILLANCE PATHWAYS

The RSR is only one of several signaling pathways that originate from distressed ribosomes. During translation, ribosomes can encounter a range of obstacles on the mRNA, such as nucleotide damage, secondary structures and lack of a stop codon. Ribosomes may also struggle with inefficient decoding due to a shortage of amino acids (98). Such events will slow down elongation and may in the most extreme case cause translational arrest of individual ribosomes. Transient pausing of the elongation cycle generally allows time for resolving such issues, however if not sufficient, quality control and stress response mechanisms are required to salvage the affected ribosomes (98–100).

### Ribosome-associated quality control

**Figure 3.**
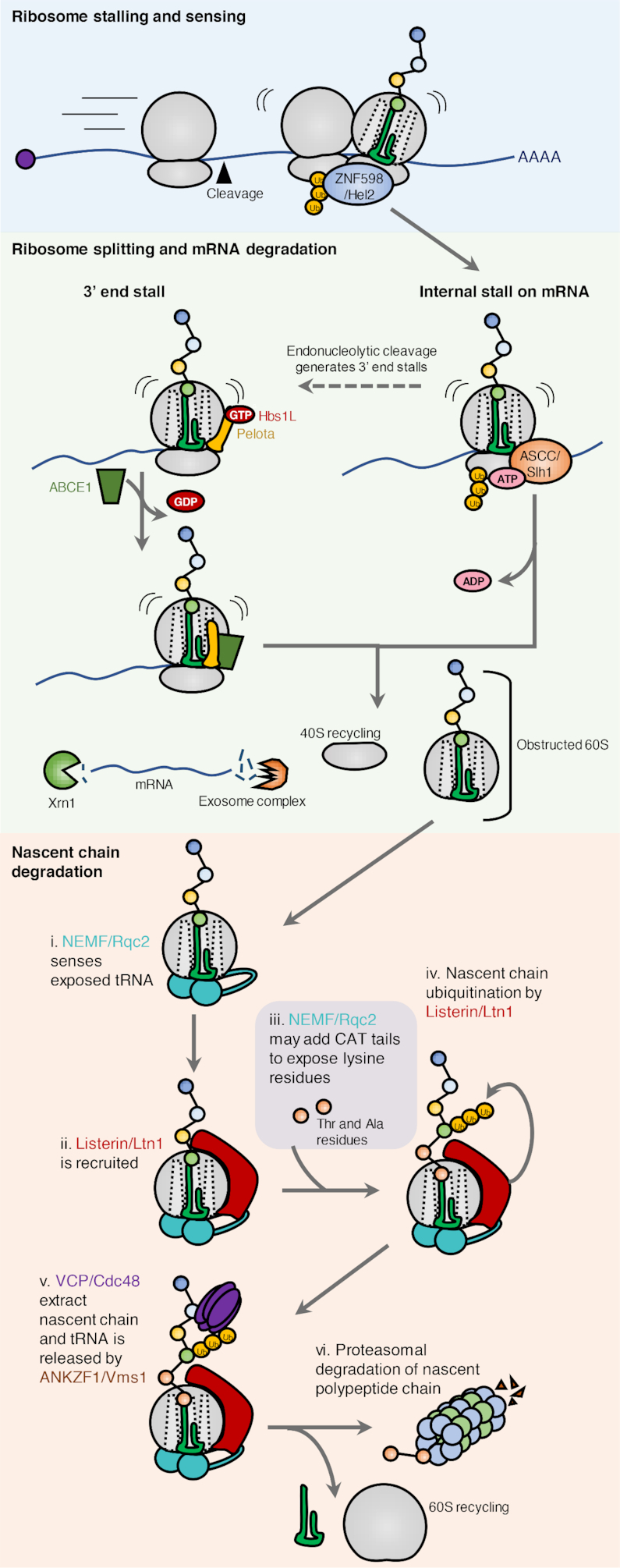
Ribosome collision and ribosome-associated quality control. (**A**) Translational stalling causes collision of ribosomes. This unique structure is recognized by the E3 ubiquitin ligase ZNF598 that promotes ubiquitination of the ribosomal proteins uS10, eS10 and uS3. Stalling on internal mRNA sequences is recognized by the ASC complex (Slh1 in yeast) which liberates the leading ribosome. The trailing ribosomes can then resume translation. Under certain circumstances, endonucleases cleave the mRNA between ribosomes resulting in ribosomes stalled on 3′end of the mRNA. This makes them accessible for splitting by the recycling factors Pelota, HBS1L and ABCE1 (Dom34, Hbs1 and Rli1 in yeast). The released mRNA is degraded by the 5′-3′ exoribonuclease Xrn1 and the exosome complex to prevent the aberrant mRNA from being translated again. While the 40S subunit is directly ready for recycling, the peptidyl-tRNA remains associated with the large ribosomal subunit. The obstructed 60S subunit is recognized by the RQC component NEMF (Rqc2 in yeast) that recruits the E3 ubiquitin ligase Listerin (Ltn1 in yeast) to the native peptide chain. NEMF/Rqc2 may employ ‘CAT-tailing’ to expose ribosome-buried and ubiquitinatable lysine residues in the native chain. Ubiquitination by listerin/Ltn1 recruits the ATPase VCP (Cdc48 in yeast), and once the nascent chain has been released from the tRNA by ANKZF1 (Vms1 in yeast), VCP can deliver the polypeptide to the proteasome, where it is degraded.

Irreversible ribosome stalling induces active splitting and rescue of ribosome subunits, and degradation of the incomplete protein product (Figure 3). Under these conditions, the ASC-1 helicase complex (Slh1/Rqt2 in yeast) or the recycling factors Pelota, HBS1L (or GTPBP2) and ABCE1 (Dom34, Hbs1 and Rli1 in yeast) split the stalled ribosome into its constituent 60S and 40S subunits (101,102). Subsequently, the released mRNA is degraded by the 5′-3′ exoribonuclease Xrn1 and the exosome complex to prevent the aberrant mRNA from recruiting a new ribosome. The released 40S subunit is recycled (reviewed in (98,100)), which likely requires the action of the recycling factor ABCE1 (103,104). The 60S subunit must first be relieved of the trapped tRNA-conjugated polypeptide chain (105).

The molecular signal for initiation of the RQC pathway involves collision of ribosomes which occurs when a trailing ribosome collides with a slowed-down or blocked leading ribosome. Such perturbations may be pathological in nature, but can also occur naturally. An example of the latter is when membrane domain insertion and/or folding fails. Decreasing the translation rate in response to inefficient folding of a transmembrane protein, can protect the cell from potentially toxic aberrant proteins (106). Collision creates a ‘disome’ with an extensive 40S–40S interface that can be recognized by different quality control factors (107–110). Recently, the very abundant EDF1 protein was reported to be the first factor to engage disomes (111,112). If collisions persist, EDF1 stabilizes the recruitment of the GIGYF2–4EHP complex and the E3 ubiquitin ligase ZNF598 (Hel2 in yeast). Both pathways aim to reduce collision events: GIGYF2–4EHP by preventing re-initiation on problematic messages, and ZNF598 by resolving the internally stalled ribosome and initiating RQC (101,113,114). ZNF598 can selectively recognize and ubiquitinate collided ribosomes, whereas GIGYF2–4EHP is dependent on EDF1 for recruitment (111). The longer stalling persist, the greater the likelihood of 40S ribosome ubiquitination by ZNF598 (110,115). It has been speculated that 40S ubiquitination signifies a ubiquitin code that distinguishes physiologically paused ribosomes from pathological structures (116). Indeed, a new study propose that the splitting factor ASCC irreversibly commits ribosomes to RQC by liberating the leading ribosome—a process that requires eS10 and uS10 ubiquitination by ZNF598 (101). In the absence of ASCC, or if the pathway is oversaturated, internally stalled ribosomes become substrates for endonuclease-mediated mRNA cleavage, likely via ubiquitin-dependent nuclease recruitment (109,117). These endonucleases sever the mRNA in between collided ribosomes, thus generating a 3′ end stall, which can be rescued by Pelota-Hbs1L-ABCE1. Consequently, absence of ubiquitination permits a certain degree of translation of aberrant mRNAs due to the lack of ASCC recruitment and inefficient mRNA cleavage (118,119).

RQC constitutes a cellular pathway for detecting and splitting stalled ribosomes, resolve the peptidyl-tRNA–60S complex and promote degradation of the nascent polypeptide chain (100,120). Resolution of the obstructed 60S subunit is initiated by NEMF (Rqc2/Tae2 in yeast), a protein that recognizes the exposed peptidyl-tRNA (121). Binding of NEMF prevents re-association of the two ribosomal subunits, as it occupies the 40S binding interphase (121). In addition, NEMF recruits the E3 ubiquitin ligase Listerin/LTN1 (Ltn1 in yeast) to ubiquitinate the nascent chain protruding from the 60S exit tunnel (118,122,123). In cases where the exposed peptide chain does not contain any lysines amenable for ubiquitination, non-canonical elongation with alanines and threonines is catalyzed by NEMF/Rqc2. This process, coined CAT-tailing, is likely to expose lysines from the part of the nascent chain that was buried within the 60S exit tunnel (124,125). Subsequently, ANKZF1 (Vms1 in yeast) releases the polypeptide from the conjugated tRNA. ANKZF1 was originally believed to be a tRNA hydrolase that attacks the peptide chain, but recent studies suggest that this enzyme instead processes the tRNA through its endonuclease activity (126,127). In the final step of RQC, the ubiquitinated nascent peptide is extracted from 60S through the ubiquitin segregase activity of p97/VCP and degraded by the proteasome (123,128).

The physiological importance of functional RQC is underscored by the fact that knockout of LTN1 is embryonically lethal. Mice carrying hypomorphic LTN1 mutations that support life suffer from early onset neurodegeneration (129). This phenotype is likely to be a consequence of compromised RQC, as mutation of the RQC factor GTPBP2, a binding partner of the ribosome recycling factor Pelota, also causes neurodegeneration in mice (130). It thus appears that RQC is an essential process that when impaired results in proteotoxic stress, including truncated and misfolded protein products, sequestration of inactivated 60S subunits, protein aggregate formation and organelle dysfunction. This will in turn contribute to proteome imbalance which is one of the hallmarks of neurodegeneration (100,131,132).

### Integrated stress response

**Figure 4.**
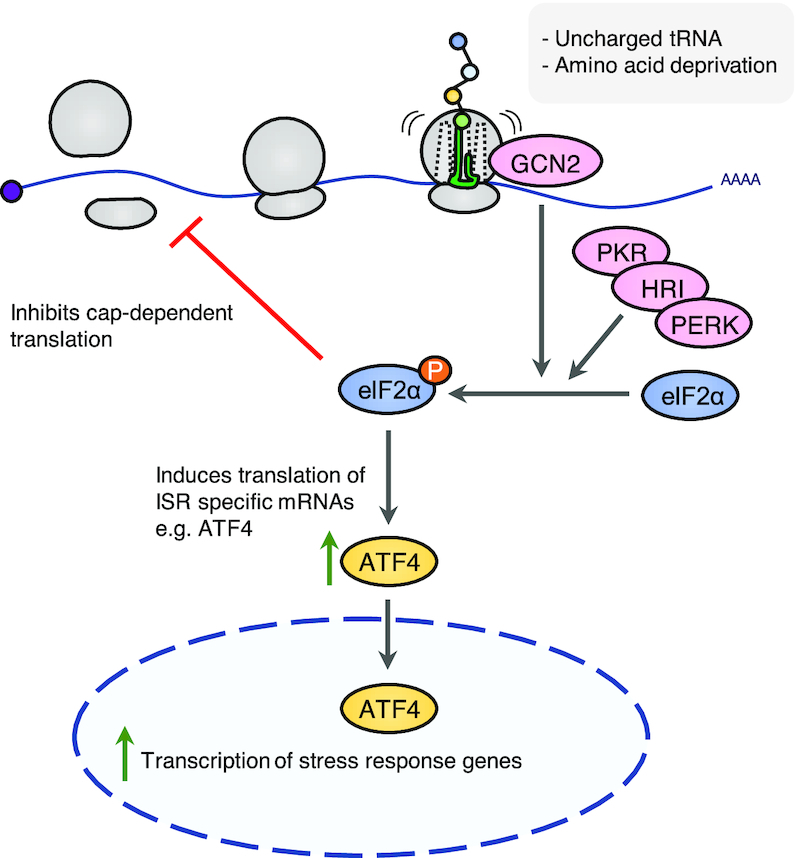
The integrated stress response. (**A**) The integrated stress response (ISR) converges on phosphorylation of eIF2α, resulting in global inhibition of cap-dependent mRNA translation. GCN2 is the relevant ISR-kinase upon translational arrest. Cap-independent pathways allow for the selective translation of ISR-specific mRNAs including ATF4. ATF4 is a transcription factor and effector of ISR that controls expression of stress response genes.

The integrated stress response is a convergence of several stress responses that respond to cellular imbalances (Figure 4). The ISR can be activated by four distinct molecular stress signals, each of which activates a specific protein kinase. Amino acid deficiency is sensed by general control non-depressible protein 2 (GCN2), heme deprivation by heme-regulated eIF2α kinase (HRI), viral infection by double stranded RNA-dependent protein kinase (PKR) and ER stress by PKR-like ER kinase (PERK) (133). Upon activation, all of these kinases phosphorylate and inhibit the function of eukaryotic translation initiation factor eIF2α (133). This phosphorylation event interferes with most forms of translation initiation, but allows the ribosome to selectively translate messages of critical stress-combatting genes, regulated by the transcription factor ATF4 (134,135). The ISR thus attempts to restore cellular homeostasis in the face of cell stress or biochemical imbalances. The ISR can also activate cell death pathways via selective translation of the pro-apoptotic transcription factors CHOP and ATF3 (133).

Shortage of amino acids and ribosome pausing are sensed by GCN2, and this is one of the triggers of the ISR (136,137). Earlier models of GCN2 activation centered around sensing of deacylated tRNAs caused by amino acid depletion. Such tRNAs were shown to be recognized by the regulatory HisRS-like domain of GCN2, located C-terminal to the kinase domain (138). In its inactive form, GCN2 is a dimer where the C-terminal segment and the HisRS-like domain engages in autoinhibitory interactions with the kinase domain *in trans*. Binding of uncharged tRNA to the HisRS-like domain *in vitro* relieves this inhibition and allows GCN2 to phosphorylate its main substrate eIF2α (139,140). *In vivo* studies in higher species have challenged this view and point towards alternative or complementary activation mechanisms for GCN2. Ishimura *et al.* observed that GCN2 is activated in brains of mice prone to widespread ribosomal stalling due to deficiency of just a single arginine tRNA isoacceptor and the ribosome rescue factor GTPBP2. The authors propose that ribosome stalling is sufficient to activate a GCN2-dependent ISR, independently of the presence of deacylated tRNAs (136).

Other recent studies lend further support and mechanistic insight into a ribosome-templated activation mechanism for GCN2 (141,142). Inglis *et al.* showed that GCN2 binds directly to ribosomes, an interaction that strongly stimulates GCN2 kinase activity *in vitro*. Binding involved both the pseudokinase domain, C-terminal Domain (CTD) and HisRS-like domains of GCN2, and was mediated by the ribosomal P-stalk. In eukaryotes, this flexible structure is a pentameric complex consisting of one subunit of the 60S ribosomal protein RPLP0 and two subunits of both RPLP1 and RPLP2. The P-stalk is situated adjacent to the ribosomal A-site and together with the SRL it forms the main ribosomal interaction site for translation elongation factors such as EEF1A and EEF2 (143). Studies in archaea show that the flexible acidic tails of P-stalk proteins RPLP1 and RPLP2 play an important role in capturing these elongation factors and direct them towards their ribosomal site of action (144,145). A potential role for the P-stalk was highlighted several years ago in yeast, where recombinant P-stalk complexes were shown to activate GCN2 *in vitro* (146). Besides corroborating that both intact ribosomes and the P-stalk activate GCN2 *in vitro*, Harding *et al.* also isolated mammalian ribosome-mutated cell lines that supported basal ribosome function, but did not allow for GCN2 activation by amino acid starvation *in vivo*. These mutations affected the P-stalk proteins, and the ability of these ribosomes to activate GCN2 *in vitro* was strongly attenuated (142). In spite of this recent insight, sensing of tRNAs may still play an important role during GCN2 activation, and it has not been clarified if the P-stalk carries a specific activating signal upon amino acid starvation or merely constitutes a structural platform on which GCN2 is activated by other signals. An attractive possibility is that the elongation factors compete with GCN2 for P-stalk binding, and thus represses GCN2 activation when the ribosome is actively translating mRNAs. This traffic is naturally interrupted by lack of amino acids and may expose the latent capacity of the P-stalk to bind and activate GCN2 (142).

GCN2 knockout mice are overall normal and present mainly with mild metabolic phenotypes, probably related to an altered response to nutrient availability. A physiological role of GCN2 in responding to amino acid deprivation was elegantly shown by Zhang *et al.*, who mated GCN2 KO female mice with heterozygous males. The genotypes of the offspring followed the expected mendelian distribution, but under conditions mimicking amino acid starvation, significantly fewer knockout pups were born and these also had a higher perinatal morbidity (147). Dysregulation of ISR signaling has been linked to tumor progression (133,148,149), and therapeutic targeting of the ISR for cancer treatment has been proposed (150,151).

## DISCUSSION

Protein translation is essential for cell survival and must be tightly controlled with respect to activity, selectivity and fidelity. The Ribotoxic Stress Response (RSR), the Ribosome-associated Quality Control (RQC) and the Integrated Stress Response (ISR) constitute a palette of surveillance pathways that monitor and respond to ribosomal dysfunction. Compared to the last two of these pathways, we know precious little about the physiological relevance of the RSR. Unlike RQC and ISR, the RSR pathway does not appear to be conserved from yeast, and the vast majority of studies on the subject address only mammalian cell lines exposed to exotic sources of ribotoxic stressors. These include ribotoxic enzymes and microbial ribosome inhibitors that cells are highly unlikely to become exposed to. It thus transpires that there must exist a more pertinent evolutionary rationale for the appearance of this stress response in multicellular organisms. Potential phenotypes of ZAK knockout mice have not yet been elucidated, and only rudimentary experiments in *C. elegans* has confirmed that this pathway supports survival and adaptation upon organismal stress (72). Resolving these issues remain pressing and is an obvious avenue for future research.

**Figure 5.**
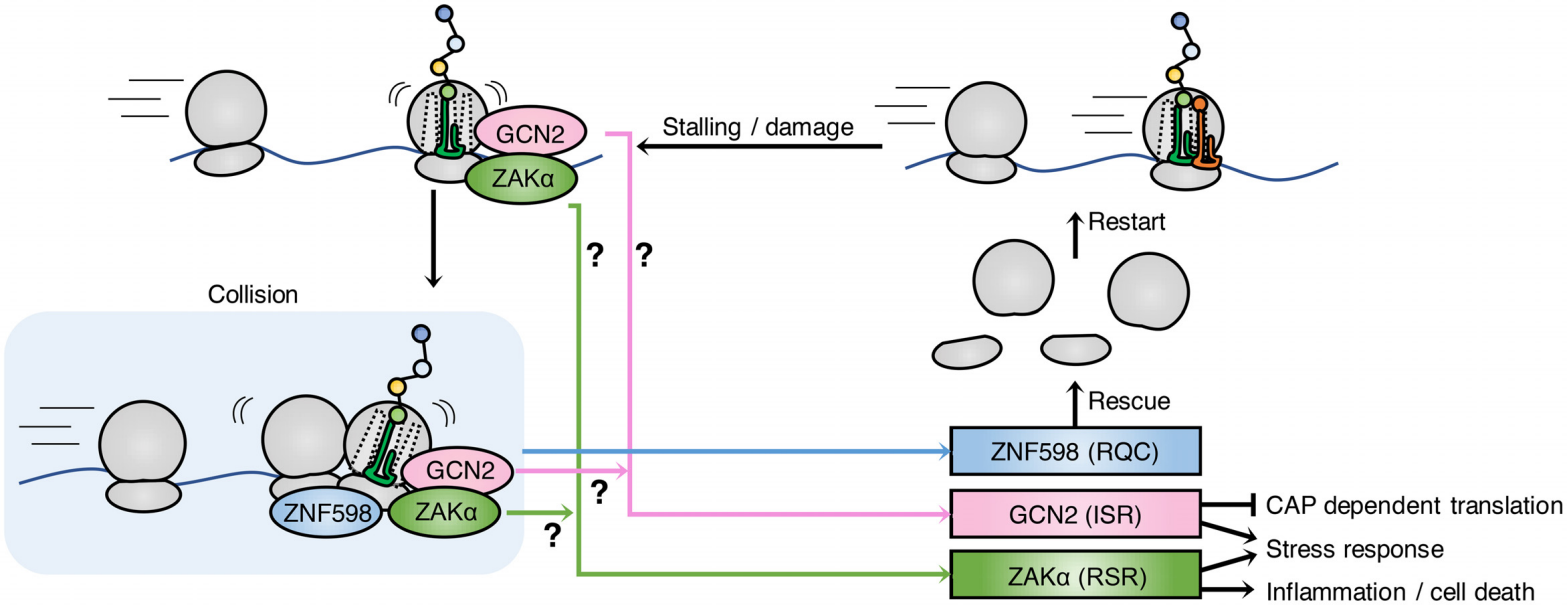
Sensing mechanisms and outcomes of ribosomal stress-surveillance pathways. Local translational arrest leads to collision of ribosomes, which is a signal for recruitment and activation of the RQC pathway sensor ZNF598. The RSR sensor ZAKα and the ISR sensor GCN2 may also be activated by recognition of this structure, or alternatively by sensing signals directly on stalled ribosomes. Once activated, the RQC pathway will attempt to rescue and recycle stalled ribosomes. The ISR pathway activates stress responses by inhibiting cap-dependent translation and facilitating selective translation of stress response proteins. The RSR pathway activates MAP kinase-driven stress responses and inflammatory signaling and may also mediate apoptotic signaling.

While the recent realization that ZAKα is a ribosome-binding and ribotoxic stress-sensing kinase is an important step forward (66,72), it also raises several unanswered questions. How can two sensing domains in a single protein accommodate the diverse structural conformations associated with the broad spectrum of ribotoxic lesions? And how are these interactions channeled into ZAKα kinase activation? The solution to this sensor conundrum does not necessarily lie in recognition of the actual lesions, but could equally well be dependent on interaction with a translation-relevant intermediate of the elongation cycle. In this scenario, ZAKα would only be competent for activation when bound to the ribosome, but kept inactive when satisfied by simultaneous probing of a non-pathological structure in the translation pocket. Recent work from the Green lab suggests that one direct, and potentially universal, activation signal for both RSR and ISR is brought about by collision of ribosomes and that a ‘disome’ structure is key for activation of ZAKα and GCN2 kinases (66) (Figure 5). In yeast, where the RSR pathway did not evolved, Gcn2 was also proposed to be activated by collision of ribosomes (66,152). This is a very interesting concept that unifies the activating signal for the three ribosomal stress responses dealt with in this review. It also suggests that RQC, RSR and ISR are physiologically interconnected, and represents a three-pronged defence against common and stochastic translational perturbations. At present, this model does not fully explain the lack of direct correlation between the potency of ribotoxic stressors with respect to inhibiting translation and activating the RSR. Nor can it account for the strongly biased activation of the RSR vs. the other stress signaling pathways by specific translation-perturbing agents (72). An attractive possibility is that collided ribosomes can present themselves in several forms that are differentially sensed by sensing factors. An alternative explanation that would satisfy the collision model is that ZAKα simultaneously monitors disome formation and other signals associated with translational arrest. Thus, while ZAKα appears to be the universal sensor of ribotoxic stress, the exact identity and full scope of ribosomal signal(s) that it senses, and how the recognition of these signals translates into kinase activation, is not clear at present.

## CONCLUDING REMARKS AND FUTURE PERSPECTIVE

We have reviewed the current knowledge on ribosomal surveillance systems with an emphasis of the roles and underlying mechanisms of RSR-associated MAP kinase signaling. Among the pathways described (RSR, RQC and ISR), our knowledge of the significance of the RSR is lacking behind. Future work is required to elucidate the physiologically relevant signals that activate this pathway and determine the full scope of its physiological roles. Given the already established connections between dysregulated RSR and pathology, such knowledge is likely to inform new therapeutic strategies for combating a range of human diseases.
